# Screening Tool to Identify Patients with Advanced Aortic Valve Stenosis

**DOI:** 10.3390/jcm11154386

**Published:** 2022-07-28

**Authors:** Sameh Yousef, Andrea Amabile, Chirag Ram, Huang Huang, Varun Korutla, Saket Singh, Ritu Agarwal, Roland Assi, Rita K. Milewski, Yawei Zhang, Prakash A. Patel, Markus Krane, Arnar Geirsson, Prashanth Vallabhajosyula

**Affiliations:** 1 Division of Cardiac Surgery, Yale School of Medicine, 330 Cedar Street BB204, New Haven, CT 06511, USA; sameh.yousef@yale.edu (S.Y.); andrea.amabile@yale.edu (A.A.); chirag.ram@yale.edu (C.R.); varun.korutla@yale.edu (V.K.); saket.singh@yale.edu (S.S.); roland.assi@yale.edu (R.A.); rita.milewski@yale.edu (R.K.M.); markus.krane@yale.edu (M.K.); arnar.geirsson@yale.edu (A.G.); 2 Section of Surgical Outcomes and Epidemiology, Yale School of Public Health, New Haven, CT 06511, USA; huang.huang@yale.edu (H.H.); yawei.zhang@yale.edu (Y.Z.); 3 Joint Data Analytics Team, Information Technology Service, Yale University, New Haven, CT 06511, USA; ritu.agarwal@ynhh.org; 4 Division of Cardiac Anesthesiology, Yale School of Medicine, New Haven, CT 06511, USA; prakash.patel@yale.edu

**Keywords:** aortic, stenosis, mortality, risk, prediction

## Abstract

(1) Background: The clinical burden of aortic stenosis (AS) remains high in Western countries. Yet, there are no screening algorithms for this condition. We developed a risk prediction model to guide targeted screening for patients with AS. (2) Methods: We performed a cross-sectional analysis of all echocardiographic studies performed between 2013 and 2018 at a tertiary academic care center. We included reports of unique patients aged from 40 to 95 years. A logistic regression model was fitted for the risk of moderate and severe AS, with readily available demographics and comorbidity variables. Model performance was assessed by the C-index, and its calibration was judged by a calibration plot. (3) Results: Among the 38,788 reports yielded by inclusion criteria, there were 4200 (10.8%) patients with ≥moderate AS. The multivariable model demonstrated multiple variables to be associated with AS, including age, male gender, Caucasian race, Body Mass Index ≥ 30, and cardiovascular comorbidities and medications. C-statistics of the model was 0.77 and was well calibrated according to the calibration plot. An integer point system was developed to calculate the predicted risk of ≥moderate AS, which ranged from 0.0002 to 0.7711. The lower 20% of risk was approximately 0.15 (corresponds to a score of 252), while the upper 20% of risk was about 0.60 (corresponds to a score of 332 points). (4) Conclusions: We developed a risk prediction model to predict patients’ risk of having ≥moderate AS based on demographic and clinical variables from a large population cohort. This tool may guide targeted screening for patients with advanced AS in the general population.

## 1. Introduction

Calcific aortic stenosis (AS) is the most common valvular heart disease in Western countries [1] and the third most common cardiovascular disease, next only to hypertension and coronary artery disease (CAD) [2]. Prevalence of moderate or severe AS increases with age, affecting 12.4% of the population aged ≥ 75 years [3]. The prevalence of the disease is also projected to increase due to the aging population and the high burden of atherosclerotic risk factors. By 2025, 0.8 million people in North America will be suffering from symptomatic severe AS, and by 2050 the number is expected to approach 1.4 million [3]. The 2-year mortality of symptomatic severe AS without intervention is >50% [4]. In addition, patients with AS have a high incidence of heart failure (HF), repeated hospitalizations, and poor quality of life [5]. Yet, there are no screening programs for such a common and morbid disease. 

AS can be readily diagnosed using Doppler echocardiography with accuracy, reasonable cost, and at no risk to the patient. Once reliably diagnosed, it can be treated effectively with interventions that improve survival and quality of life [6,7]. In the absence of a screening program, many patients are diagnosed late in the course of the disease, mainly because of the reliance on symptom appearance to prompt testing [8]. The disease typically runs an asymptomatic course. Even patients with severe AS are frequently asymptomatic or endorse non-specific symptoms [9,10]. These challenging aspects of AS have led to under-diagnosis and consequently under-treatment of the disease with devastating consequences for the lethality and morbidity of the disease. Hence, there is a need for tools that guide screening programs for early diagnosis in various settings (cardiology offices, primary care offices, online health clinics, etc.) and timely treatment of AS patients. 

Previous efforts have not been sufficient to reliably characterize AS risk factors and successfully use them to generate a risk score for general screening recommendations to identify patients with moderate or severe AS. In particular, this was due to the small size of the available cohorts and/or the lack of control groups. Towards this end, we leveraged a large echocardiography database to develop a prediction model to identify patients with AS based on readily available demographic and comorbidity information. 

## 2. Materials and Methods

### 2.1. Patient Population and Data Source

This cross-sectional study was conducted at Yale-New Haven Hospital, a tertiary care center in the United States, serving the community of the greater New Haven area and a large fraction of the population of the state of Connecticut (with multiple ethnic backgrounds, age groups, and comorbidity profiles). Echocardiography data and electronic health records (EHR) were queried for all patients ≥ 18 years old who had at least one study during the calendar years 2013–2018. These criteria yielded 146,876 studies obtained on 48,524 unique patients. The Institutional Review Board at Yale University approved this study, and individual consent was waived.

### 2.2. Analytic Cohort Building

**Figure 1:**
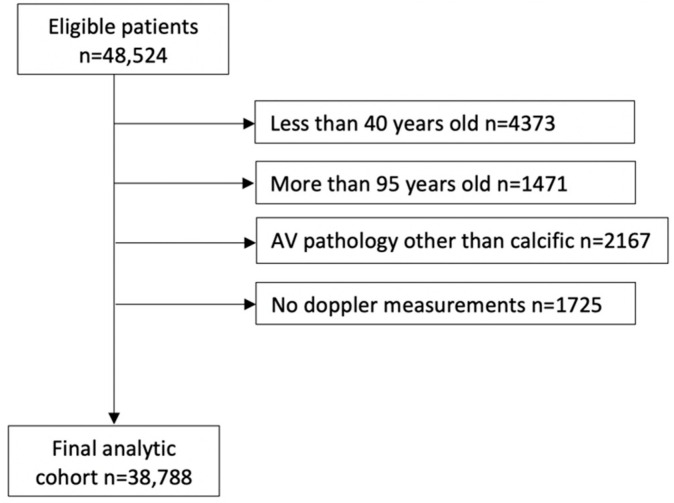
Analytic cohort building. Consort diagram describing the inclusion and exclusion steps that led to the final analytic cohort.

A series of exclusions led to the final analytic cohort of 38,788 patients, as shown in Figure 1. Given that AS is rare before age 40, patients less than 40 years old at the time of their initial study were excluded. Patients more than 95 years old at the time of their initial study were also excluded because intervention adds minimal benefit to longevity at this old age. We also excluded patients with prosthetic aortic valves (AV) on their initial echocardiography during the study period, patients with AV pathology other than calcific AS (rheumatic AS, endocarditis, HOCM, moderate and severe AI, and aortic valve tumor), patients who had aortic valve replacement (AVR) as part of aortic aneurysm or dissection repair, and patients who received heart transplantation or ventricular assist device treatment. Studies missing all AV Doppler parameters were also excluded. We intentionally excluded patients with mild AS from the models because the focus of this analysis was to develop a screening algorithm to help identify patients with ≥moderate AS.

### 2.3. Moderate and Severe AS

Patients with ≥ moderate AS were identified using the Doppler echocardiography parameters clinically used to define AS. The most severe value of the following parameters was considered [11]: 

Aortic valve area (AVA) ≤ 1.5 cm^2^ and or dimensionless valve index (DVI) ≤ 0.5 and or maximum flow velocity (V-max) ≥ 3 m/s, and or mean pressure gradient (PG-mean) ≥ 20 mmHg.

### 2.4. Predictor Variables

Candidate variables included demographics (age, sex, and race), clinical characteristics (body mass index, smoking history, and other comorbidities that are common in this age group), medications (beta blocking agents, calcium channel blocking agents, diuretics, angiotensin-converting enzyme pathway inhibitors/blockers, aspirin, and statins), and previous interventions including coronary artery bypass graft (CABG), percutaneous coronary intervention (PCI), defibrillator and/or pacemaker implantation. Demographics, BMI, history of smoking, and medications were directly abstracted from the EHR. Comorbidities and interventions were queried in the form of ICD-10 codes (Appendix A).

### 2.5. Variable Selection and Model Development

Distribution of patient demographics, comorbidity, and medications between patients with ≥moderate AS and those with no AS were compared by Student’s t-test for continuous variables and by chi-squared test for categorical variables, respectively. Association between the risk of moderate/severe AS and patient demographics, comorbidity, and medications were evaluated using the multivariable logistic regression. The variables included in the regression model were chosen based on stepwise variable selection, optimizing the Akaike Information Criterion (AIC) value.

### 2.6. Performance Metrics

We evaluated the model discrimination ability using the area under the receiver-operator characteristics curve (AUROC), which characterizes model discrimination and ranges between 0 and 1, with a higher value corresponding to better discrimination. AUROC is the proportion of the times patients with an event were accurately classified to have a higher probability of event within all possible pairs of patients with and without an event. Calibration was characterized using continuous calibration plot.

### 2.7. Clinical Interpretability

To enhance clinical implementation of the models in assessing patients’ risk of having the disease and, accordingly, the need for echocardiographic testing, we further developed a simple integer scoring system based on the β coefficients for each predictor variable in the logistic regression models. A two-tailed *p* < 0.05 was considered statistically significant. All analyses were carried out using SAS 9.4 (SAS Institute, Cary, NC, USA).

## 3. Results

A series of exclusions led to the final analytic cohort of 38,788 patients (Figure 1). Among them, 4200 (10.8%) patients met the criteria for ≥moderate AS. On unadjusted comparisons, patients with ≥moderate AS, compared to patients with no AS, were older (mean age: 76.6 ± 11.3 vs. 66.4 ± 13.3 years, *p* < 0.0001), more likely to be males, Caucasian race, with higher prevalence of hypertension, dyslipidemia, coronary artery disease (CAD), pulmonary hypertension, heart block (HB), atrial fibrillation (AF), cerebral infarction, peripheral vascular disease (PVD), chronic kidney disease (CKD), diabetes mellitus (DM), dementia, inability to walk, heart failure (HF), dilated cardiomyopathy, pacemaker, defibrillator, PCI, and CABG and were more likely to be on beta-blockers, CCBs, ACEIs, and diuretics (Table 1).

In the multivariable model, the risk of having moderate and severe AS, in reference to no AS, was associated with increasing age (OR = 1.055 CI = 1.051–1.059 for every one-year increment from 40), use of aspirin (OR = 1.21, CI = 1.11–1.31), use of CCBs (OR = 1.2, CI = 1.12–1.29), use of diuretics (OR = 1.58, CI = 1.46–1.7), CABG (OR = 1.37, CI = 1.22–1.53), CAD (OR = 1.14, CI = 1.05–1.24), dyslipidemia (OR = 1.23, CI = 1.13–1.32), pulmonary hypertension (OR = 1.69, CI = 1.39–2.04), cardiomyopathy (OR = 1.74, CI = 1.12–2.71), HB (OR = 1.31, CI = 1.14–1.5), AF (OR = 1.15, CI = 1.06–1.24), CVD (OR = 1.24, CI = 1.02–1.5), PVD (OR = 1.29, CI = 1.14–1.46), CKD (OR = 1.2, CI = 1.09–1.33), DM (OR = 1.08, CI = 1.00–1.16), chronic liver disease (OR = 1.64, CI = 1.38–1.97), history of obesity (OR = 1.21, CI = 1.06–1.39), pacemaker (OR = 1.2, CI = 1.01–1.42), and heart failure (OR = 1.39, CI = 1.26–1.52). The risk of having moderate or severe AS decreased with African American race (AA) (OR = 0.5, CI = 0.44–0.56), races other than Caucasian and AA (OR = 0.83, CI = 0.72–0.95) and history of dementia (OR = 0.7, CI = 0.6–0.81) (Table 2).

**Figure 2:**
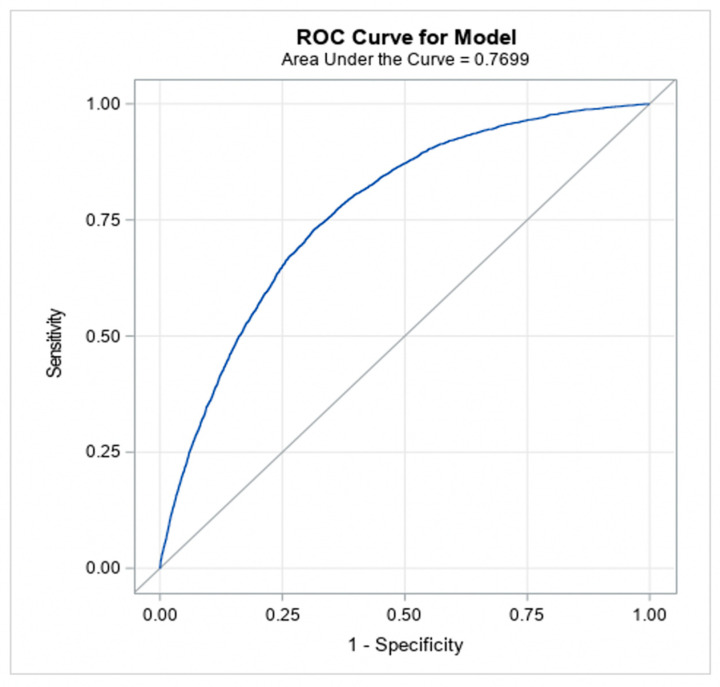
Discrimination ability of the model (to classify patients into moderate/severe AS versus no AS).

**Figure 3:**
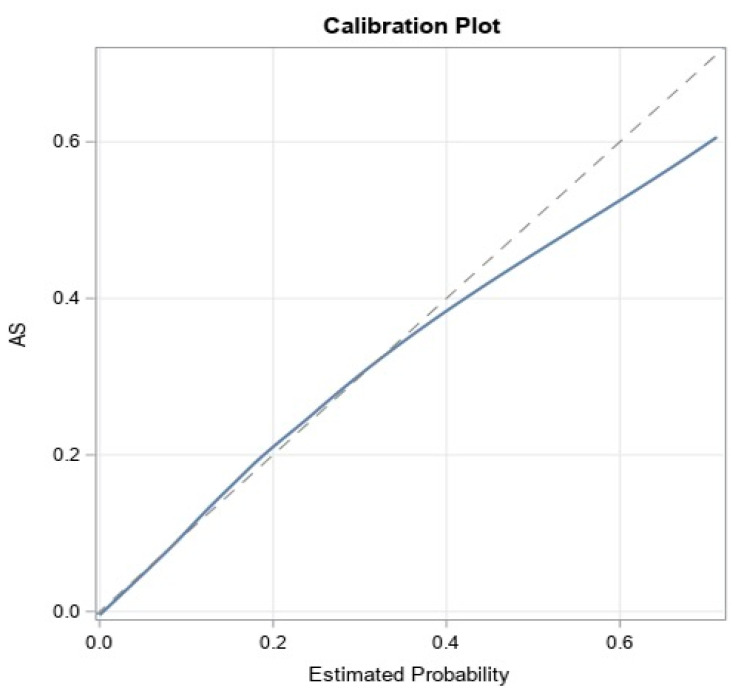
Calibration of the model.

The models achieved good predictive discrimination with an AUC of 0.77 (Figure 2) and were well calibrated by visual examination of the calibration plots (Figure 3). The coefficients were transformed to risk scores that can be translated into the probability of individual patient having ≥moderate AS (Table 3). The risk scores assigned to each risk factor are summarized in a nomogram, which also shows translation between the overall points and the predicted probability of ≥moderate AS (Table 4).

## 4. Discussion

The current lack of clinical tools to facilitate targeted screening of the population for patients with increased risk of ≥moderate AS is concerning given the increasing burden of the disease and its association with poor outcomes when diagnosed late or left untreated. Furthermore, timely intervention in this subpopulation can lead to significantly improved quality of life and survival, and therefore, a reliable screening program for AS would have an impact on patient outcomes. This contrasts with other conditions such as abdominal aortic aneurysm and lung cancer, for which successful implementation of nationwide screening programs led to the mitigation of their consequences [12,13,14]. Given that the prevalence of AS is much higher than these conditions for which there are screening programs, the necessity for the development of a screening tool for AS is warranted. To facilitate systematic screening of AS, we developed a model to predict a patient’s risk of having moderate/severe AS based on clinical parameters. The model is based on readily available demographic and comorbidity information that was further integrated into an integer scoring system that can be used to predict patients’ risk of having AS via the simple addition of points. 

Many of the risk factors that showed the importance in the model are shared by other atherosclerotic diseases such as CAD and PAD. According to the model, the risk of advanced AS incrementally increased after age 40, although fast progression and earlier disease onset have been shown in certain groups, including patients with bicuspid aortic valve and patients with chronic kidney disease [15,16]. Advanced AS (i.e., ≥moderate) was also associated with male gender and Caucasian race, and the association with gender was less prominent compared to the association with race as shown by the ß-estimates. This agrees with the report by Patel et al., where AA patients were less likely to have severe AS (OR = 0.41) [17]; and the report by Owens et al. from the Multi-Ethnic Study of Atherosclerosis that showed that the risk of progression of AV calcification was associated with male sex [18] The integer scoring system that we developed in this model collectively calculates the risk of AS based on multiple factors rather than considering one specific risk factor. For example, an AA woman (theoretically least likely to develop moderate/severe AS) may score up to 332 points, which corresponds to a risk of 0.6.

The model has the potential to be a valuable tool in clinical practice, given its simplification to an integer (points) system and reliance on variables that are readily available in most EHR systems. Yet, implementation of the model in a clinical setting should start with caution as the model has not been validated in prospective studies. The results of the model do not show a certain threshold at which screening should be recommended. A score of 207–235 (predicted risk of 5–10%) can be used as a threshold in the beginning, and further validating studies can help fine-tune it. Starting with a low threshold is reasonable considering the high prevalence of AS and the safety profile of echocardiography without contrast exposure. Additionally, echocardiography as a screening test for AS can be valuable as a screening test for other diseases that are prevalent in this population, such as thoracic aortic aneurysms, by providing information on aortic size. 

### Limitations

This study has some limitations. It is a single-center study, which might limit the generalizability of the results, even though the cohort is very large and included patients from nearly all backgrounds. However, the model has not been externally validated, and its implication in clinical settings requires caution until its validity is proven by other studies. The study included all comers with ≥moderate AS and the history of previously diagnosed versus incidental finding of AS is unknown, and the effect of such information on the analysis is uncertain. Finally, the study relied on claims data (ICD-10 codes) to assess the presence or absence of comorbidities, and such data are reported to be subject to over- or under-reporting.

## 5. Conclusions

We developed a risk model to identify patients aged between 40 and 95 years who are at increased risk of having ≥moderate AS based on demographic and clinical characteristics. Discrimination and calibration of the model were good. This prediction rule may guide the targeted screening of patients at increased risk of having advanced AS.

## Figures and Tables

**Table 1:** Distribution of the potential predictors between patients with advanced AS and those without AS.

Potential Predictor			No AS (*n* = 31,304)		Moderate/Severe AS (*n* = 4200)		
			Mean/Number	SD/%	Mean/Number	SD/%	*p*-Value
Age (years)			66.4	13.3	76.6	11.3	<0.0001
Gender							
	Male		15,935	50.9	2220	52.9	
	Female		15,369	49.1	1980	47.1	0.017
Race/ethnicity							
	Caucasian		22,711	72.6	3607	85.9	
	African American		5460	17.4	324	7.7	
	Other race		3133	10.0	269	6.4	<0.0001
BMI (kg/m^2^)							
	<18.5		1258	4.0	153	3.6	
	18.5–24.9		8921	28.5	1378	32.8	
	25–29.9		9482	30.3	1260	30.0	
	≥30		11,475	36.7	1409	33.6	<0.0001
Smoking							
	No		21,828	69.7	2947	70.2	
	Yes		9476	30.3	1253	29.8	0.56
Medication							
	Aspirin						
		No	8801	28.1	1110	26.4	
		Yes	16,834	53.8	2877	68.5	
		Missing	5669	18.1	213	5.1	<0.0001
	Statins						
		No	10,336	33.0	1207	28.7	
		Yes	15,299	48.9	2780	66.2	
		Missing	5669	18.1	213	5.1	<0.0001
	Beta-blockers						
		No	17,073	54.5	1663	39.6	
		Yes	14,231	45.5	2537	60.4	<0.0001
	CCB						
		No	20,346	65.0	2253	53.6	
		Yes	10,958	35.0	1947	46.4	<0.0001
	ACEI						
		No	23,077	73.7	2972	70.8	
		Yes	8227	26.3	1228	29.2	<0.0001
	Diuretic						
		No	17,593	56.2	1383	32.9	
		Yes	13,711	43.8	2817	67.1	<0.0001
Comorbidity							
	CABG						
		No	29,445	94.1	3565	84.9	
		Yes	1859	5.9	635	15.1	<0.0001
	PCI						
		No	28,762	91.9	3694	88.0	
		Yes	2542	8.1	506	12.1	<0.0001
	Hypertension						
		No	10,977	35.1	899	21.4	
		Yes	20,327	64.9	3301	78.6	<0.0001
	CAD						
		No	23,597	75.4	2461	58.6	
		Yes	7707	24.6	1739	41.4	<0.0001
	Dyslipidemia						
		No	17,266	55.2	1683	40.1	
		Yes	14,038	44.8	2517	59.9	<0.0001
	Pulmonary hypertension						
		No	30,734	98.2	4031	96.0	
		Yes	570	1.8	169	4.0	<0.0001
	Cardiomyopathy						
		No	31,192	99.6	4172	99.3	
		Yes	112	0.4	28	0.7	0.0027
	HB						
		No	30,131	96.3	3858	91.9	
		Yes	1173	3.8	342	8.1	<0.0001
	Cardiac arrest						
		No	31,173	99.6	4178	99.5	
		Yes	131	0.4	22	0.5	0.33
	AF						
		No	26,161	83.6	2912	69.3	
		Yes	5143	16.4	1288	30.7	<0.0001
	ICH						
		No	30,877	98.6	4163	99.1	
		Yes	427	1.4	37	0.9	0.0096
	Cerebral infarction						
		No	27,631	88.3	3621	86.2	
		Yes	3673	11.7	579	13.8	0.0001
	CVD						
		No	30,657	97.9	4046	96.3	
		Yes	647	2.1	154	3.7	<0.0001
	PVD						
		No	29,756	95.1	3746	89.2	
		Yes	1548	5.0	454	10.8	<0.0001
	Chronic lung disease						
		No	24,087	77.0	3178	75.7	
		Yes	7217	23.1	1022	24.3	0.065
	CKD						
		No	28,512	91.1	3526	84.0	
		Yes	2792	8.9	674	16.1	<0.0001
	DM						
		No	23,016	73.5	2805	66.8	
		Yes	8288	26.5	1395	33.2	<0.0001
	Chronic liver disease						
		No	30,135	96.3	4034	96.1	
		Yes	1169	3.7	166	4.0	0.49
	Dementia						
		No	30,139	96.3	3975	94.6	
		Yes	1165	3.7	225	5.4	<0.0001
	Frailty						
		No	30,902	98.7	4155	98.9	
		Yes	402	1.3	45	1.1	0.25
	Inability to walk						
		No	30,946	98.9	4134	98.4	
		Yes	358	1.1	66	1.6	0.017
	Depression						
		No	26,505	84.7	3690	87.9	
		Yes	4799	15.3	510	12.1	<0.0001
	Malnutrition						
		No	31,213	99.7	4192	99.8	
		Yes	91	0.3	8	0.2	0.25
	Obesity						
		No	28,989	92.6	3885	92.5	
		Yes	2315	7.4	315	7.5	0.8100
	Pacemaker						
		No	30,657	97.9	3984	94.9	
		Yes	647	2.1	216	5.1	<0.0001
	Defibrillator						
		No	30,688	98.0	4078	97.1	
		Yes	616	2.0	122	2.9	<0.0001
	HF						
		No	28,373	90.6	3333	79.4	
		Yes	2931	9.4	867	20.6	<0.0001

BMI—body mass index; CCB—calcium channel blockers; ACEIS—angiotensin-converting enzyme inhibitors; CABG—coronary artery bypass graft; PCI—percutaneous coronary interventions; CAD—coronary artery disease; HB—heart block; AF—atrial fibrillation; ICH—intra-cranial hemorrhage; CVD—cerebrovascular disease; PVD—peripheral vascular disease; CKD—chronic kidney disease; DM—diabetes mellitus; HF—heart failure.

**Table 2:** Logistic regression coefficients and ORs for advanced AS.

Predictor			Moderate/Severe AS			
			β Coefficient	*p*-Value	OR	95% CI
Age (every 1-year increment from 40)			0.0535	<0.0001	1.055	1.051–1.059
Gender						
	Male		0.0862	0.019	1.09	1.01–1.17
	Female		ref.		1.00	
Race/ethnicity						
	Caucasian		ref.		1.00	
	African American		−0.7024	<0.0001	0.50	0.44–0.56
	Others		−0.1900	0.0067	0.83	0.72–0.95
BMI (kg/m^2^)						
	<18.5		−0.2033	0.033	0.82	0.68–0.98
	18.5–24.9		ref.		1.00	
	25–29.9		−0.0106	0.81	0.99	0.91–1.08
	≥30		0.1326	0.0048	1.14	1.04–1.25
Medication						
	Aspirin					
		No	ref.		1.00	
		Yes	0.1896	<0.0001	1.21	1.11–1.31
	Statins					
		No	ref.		1.00	
		Yes	0.0839	0.055	1.09	1.00–1.19
	CCB					
		No	ref.		1.00	
		Yes	0.1844	<0.0001	1.20	1.12–1.29
	Diuretic					
		No	ref.		1.00	
		Yes	0.4545	<0.0001	1.58	1.46–1.70
Comorbidity						
	CABG					
		No	ref.		1.00	
		Yes	0.3140	<0.0001	1.37	1.22–1.53
	HTN					
		No	ref.		1.00	
		Yes	0.0806	0.074	1.08	0.99–1.18
	CAD					
		No	ref.		1.00	
		Yes	0.1301	0.0022	1.14	1.05–1.24
	Dyslipidemia					
		No	ref.		1.00	
		Yes	0.2028	<0.0001	1.23	1.13–1.32
	Pulmonary hypertension					
		No	ref.		1.00	
		Yes	0.5233	<0.0001	1.69	1.39–2.04
	Cardiomyopathy					
		No	ref.		1.00	
		Yes	0.5537	0.015	1.74	1.12–2.71
	Heart block					
		No	ref.		1.00	
		Yes	0.2679	0.0001	1.31	1.14–1.50
	AF					
		No	ref.		1.00	
		Yes	0.1367	0.0009	1.15	1.06–1.24
	ICH					
		No	ref.		1.00	
		Yes	−0.4918	0.0062	0.61	0.43–0.87
	CVD					
		No	ref.		1.00	
		Yes	0.2135	0.029	1.24	1.02–1.50
	PVD					
		No	ref.		1.00	
		Yes	0.2552	<0.0001	1.29	1.14–1.46
	CKD					
		No	ref.		1.00	
		Yes	0.1836	0.0004	1.20	1.09–1.33
	DM					
		No	ref.		1.00	
		Yes	0.0735	0.066	1.08	1.00–1.16
	Chronic liver disease					
		No	ref.		1.00	
		Yes	0.4972	<0.0001	1.64	1.38–1.97
	Dementia					
		No	ref.		1.00	
		Yes	−0.3608	<0.0001	0.70	0.60–0.81
	Obesity					
		No	ref.		1.00	
		Yes	0.1929	0.0063	1.21	1.06–1.39
	Pacemaker					
		No	ref.		1.00	
		Yes	0.1799	0.040	1.20	1.01–1.42
	HF					
		No	ref.		1.00	
		Yes	0.3256	<0.0001	1.39	1.26–1.52

BMI—body mass index; CCB—calcium channel blockers; ACEIS—angiotensin-converting enzyme inhibitors; CABG—coronary artery bypass graft; PCI—percutaneous coronary interventions; CAD—coronary artery disease; HB—heart block; AF—atrial fibrillation; ICH—intra-cranial hemorrhage; CVD—cerebrovascular disease; PVD—peripheral vascular disease; CKD—chronic kidney disease; DM—diabetes mellitus; HF—heart failure; HTN—hypertension.

**Table 3:** Score prediction for ≥ moderate AS.

Predictor			Moderate/Severe AS		
			β Coefficient	Linear Predictor	Point
Age (every 1-year increment from 40)			0.0535		
	40–49			0.0000	0.0
	50–59			0.5350	20.0
	60–69			1.0700	40.0
	70–79			1.6050	60.0
	80–89			2.1400	80.0
	90+			2.6750	100.0
Gender					
	Male		0.0862	0.0862	3.2
	Female		ref.	0.0000	0.0
Race/ethnicity					
	Caucasian		0.7024	0.7024	26.3
	African American		ref.	0.0000	0.0
	Others		0.5125	0.5125	19.2
BMI (kg/m^2^)					
	<18.5		ref.	0.0000	0.0
	18.5–24.9		0.2033	0.2033	7.6
	25–29.9		0.1926	0.1926	7.2
	≥30		0.3359	0.3359	12.6
Medication					
	Aspirin				
		No	ref.	0.0000	0.0
		Yes	0.1896	0.1896	7.1
	Statins				
		No	ref.	0.0000	0.0
		Yes	0.0839	0.0839	3.1
	CCB				
		No	ref.	0.0000	0.0
		Yes	0.1844	0.1844	6.9
	Diuretic				
		No	ref.	0.0000	0.0
		Yes	0.4545	0.4545	17.0
Comorbidity					
	CABG				
		No	ref.	0.0000	0.0
		Yes	0.3140	0.3140	11.7
	Hypertension				
		No	ref.	0.0000	0.0
		Yes	0.0806	0.0806	3.0
	CAD				
		No	ref.	0.0000	0.0
		Yes	0.1301	0.1301	4.9
	Dyslipidemia				
		No	ref.	0.0000	0.0
		Yes	0.2028	0.2028	7.6
	Pulmonary hypertension				
		No	ref.	0.0000	0.0
		Yes	0.5233	0.5233	19.6
	Dilated cardiomyopathy				
		No	ref.	0.0000	0.0
		Yes	0.5537	0.5537	20.7
	HB				
		No	ref.	0.0000	0.0
		Yes	0.2679	0.2679	10.0
	AFIB				
		No	ref.	0.0000	0.0
		Yes	0.1367	0.1367	5.1
	ICH				
		No	0.4918	0.4918	18.4
		Yes	ref.	0.0000	0.0
	CVD				
		No	ref.	0.0000	0.0
		Yes	0.2135	0.2135	8.0
	PVD				
		No	ref.	0.0000	0.0
		Yes	0.2552	0.2552	9.5
	CKD				
		No	ref.	0.0000	0.0
		Yes	0.1836	0.1836	6.9
	DM				
		No	ref.	0.0000	0.0
		Yes	0.0735	0.0735	2.7
	Chronic liver disease				
		No	ref.	0.0000	0.0
		Yes	0.4972	0.4972	18.6
	Dementia				
		No	0.3608	0.3608	13.5
		Yes	ref.	0.0000	0.0
	Obesity				
		No	ref.	0.0000	0.0
		Yes	0.1929	0.1929	7.2
	Pacemaker				
		No	ref.	0.0000	0.0
		Yes	0.1799	0.1799	6.7
	HF				
		No	ref.	0.0000	0.0
		Yes	0.3256	0.3256	12.2

BMI—body mass index; CCB—calcium channel blockers; CABG—coronary artery bypass graft; PCI—percutaneous coronary interventions; CAD—coronary artery disease; HB—heart block; AFIB—atrial fibrillation; ICH—intra-cranial hemorrhage; CVD—cerebrovascular disease; PVD—peripheral vascular disease; CKD—chronic kidney disease; DM—diabetes mellitus; HF—heart failure.

**Table 4:** Linear project to the risk probability and total score.

Moderate/Severe AS		
Risk Probability	Linear Predictor	Total Score
0.0002	−8.4803 (min)	0
0.01	−4.5951	145
0.05	−2.9444	207
0.10	−2.1972	235
0.15	−1.7346	252
0.20	−1.3863	265
0.25	−1.0986	276
0.30	−0.8473	285
0.35	−0.6190	294
0.40	−0.4055	302
0.45	−0.2007	310
0.50	0.0000	317
0.55	0.2007	325
0.60	0.4055	332
0.65	0.6190	340
0.70	0.8473	349
0.75	1.0986	358
0.7711	1.2147 (max)	362
Linear predictor for reference level		−8.4803 (β0)
Minimum linear predictor		−8.4803
Maximum linear predictor		1.2147
Scores per unit of linear predictor		37.3832
Linear predictor units per score		0.0268

## Data Availability

Not applicable.

## Appendix A

**Table A1:** ICD-10 code used to define comorbidities.

Hypertension	I10, I11, I11.0, I11.9, I12, I12.0, I12.9, I13, I13.0, I13.1, I13.10, I13.11, I13.2, I15, I15.0, I15.1, I15.2, I15.8, I15.9
Coronary Heart Disease	I20, I20.0, I20.1, I20.8, I20.9, I21, I21.0, I21.01, I21.02, I21.09, I21.1, I21.11, I21.19, I21.2, I21.21, I21.29, I21.3, I21.4, I21.9, I24.8, I24.9, I25, I25.1, I25.10, I25.11, I25.11, I25.110, I25.111, I25.118, I25.119, I25.2, I25.3, I25.5, I25.6, I25.8, I25.81, I25.82, I25.83, I25.84, I25.89
Dyslipidemia	E78, E78.0, E78.00, E78.01, E78.1, E78.2, E78.3, E78.4, E78.41, E78.49, E78.5, E78.6
Pulmonary Hypertension	I27.0, I27.2, I27.20, I27.21, I27.22, I27.23, I27.24, I27.29, I27.81
Endocarditis	I33.0, I33.9, I38, I39
Mitral Regurgitation	I34.0, I34.1, I34.8, I34.9
Aortic Stenosis	I35.0, I35.2
Tricuspid Regurgitation	I36.1, I36.8, I36.9
Cardiomyopathy	I42.0, I42.1, I42.2
Heart Block	I44.0, I44.1, I44.2, I44.30, I44.39, I44.4, I44.5, I44.60, I44.69, I44.7, I45.0, I45.10, I45.19, I45.2, I45.3, I45.4, I45.6, I45.81, I45.89, I45.9
Cardiac Arrest	I46.2, I46.8, I46.9
Atrial Fibrillation	I48.0, I48.1, I48.11, I48.19, I48.2, I48.20, I48.21, I48.91, I48.3, I48.4, I48.92, I49.5
Heart Failure	I11.0, I13.0, I13.1, I13.10, I13.11, I13.2, I50.1, I50.2, I50.20, I50.21, I50.22, I50.23, I50.3, I50.30, I50.31, I50.32, I50.33, I50.4, I50.40, I50.41, I50.42, I50.43, I50.8, I50.81, I50.810, I50.811, I50.812, I50.813, I50.814, I50.82, I50.84, I50.9
Stroke	I60, I60.0, I60.00, I60.01, I60.02, I60.1, I60.10, I60.11, I60.12, I60.2, I60.3, I60.30, I60.31, I60.32, I60.4, I60.5, I60.50, I60.51, I60.52, I60.6, I60.6, I60.7, I60.8, I60.9, I61, I61.0, I61.1, I61.2, I61.3, I61.4, I61.5, I61.6, I61.8, I61.9, I62, I62.0, I62.00, I62.01, I62.02, I62.03, I62.1, I62.9, I63, I63.0, I63.00, I63.01, I63.011, I63.012, I63.013, I63.019, I63.03, I63.031, I63.032, I63.033, I63.039, I63.09, I63.1, I63.10, I63.11, I63.111, I63.112, I63.113, I63.119, I63.12, I63.13, I63.131, I63.132, I63.133, I63.139, I63.19, I63.2, I63.20, I63.21, I63.211, I63.212, I63.213, I63.219, I63.22, I63.23, I63.231, I63.232, I63.233, I63.239, I63.29, I63.3, I63.30, I63.31, I63.311, I63.312, I63.313, I63.319, I63.32, I63.321, I63.322, I63.323, I63.329, I63.33, I63.331, I63.332, I63.333, I63.339, I63.34, I63.341, I63.342, I63.343, I63.349, I63.39, I63.4, I63.40, I63.41, I63.411, I63.412, I63.413, I63.419, I63.42, I63.421, I63.422, I63.423, I63.429, I63.43, I63.431, I63.432, I63.433, I63.439, I63.44, I63.441, I63.442, I63.443, I63.449, I63.49, I63.5, I63.50, I63.51, I63.511, I63.512, I63.513, I63.519, I63.52, I63.521, I63.522, I63.523, I63.529, I63.53, I63.531, I63.532, I63.533, I63.539, I63.54, I63.541, I63.542, I63.543, I63.549, I63.59, I63.6, I63.8, I63.81,I63.89, I63.9
Cerebrovascular Disease	I65.0, I65.01, I65.02, I65.03, I65.09, I65.1, I65.2, I65.21, I65.22, I65.23, I65.29, I65.8, I66, I66.0, I66.01, I66.02, I66.03, I66.09, I66.1, I66.11, I66.12, I66.13, I66.19, I66.2, I66.21, I66.22, I66.23, I66.29, I66.3, I66.8, I66.9, I67, I67.0, I67.1, I67.2, I67.3, I67.4, I67.5, I67.6, I67.7, I67.8, I67.81, I67.82, I67.83, I67.84, I67.9
Peripheral Vascular Disease	I73.9, I73.89, E08.5, E09.5, E10.5, E11.5, E13.5, I70.20, I70.21, I70.22, I70.26, I70.29, I70.23, I70.24, I70.25, I70.211, I70.212, I70.213, I70.218, I70.311, I70.312, I70.313, I70.318, I70.611
Chronic Lung Disease	J40, J41, J41.0, J41.1, J41.8, J42, J43, J43.9, J44, J44.0, J44.1, J44.9, J45, J45.2, J45.20, J45.21, J45.22, J45.3, J45.30, J45.31, J45.32, J45.4, J45.40, J45.41, J45.42, J45.5, J45.50, J45.51, J45.52, J45.9, J45.90, J45.901, J45.902, J45.909, J45.99, J45.990, J45.991, J45.998, J47, J47.0, J47.1, J47.9
Chronic Kidney Disease	N18, N18.1, N18.2, N18.3, N18.4, N18.5, N18.6, N18.9, N19
Diabetes Mellitus	E10, E11, E11.2, E11.21, E11.22, E11.29, E11.3, E11.31, E11.39, E11.32, E11.321, E11.329, E11.33, E11.331, E11.339, E11.34, E11.341, E11.349, E11.35, E11.351, E11.352, E11.353, E11.354, E11.355, E11.359, E11.36, E11.37, E11.39, E11.4, E11.40, E11.41, E11.42, E11.43, E11.44, E11.49, E11.5, E11.51, E11.52, E11.59, E11.6, E11.61, E11.610, E11.618, E11.62, E11.620, E11.621, E11.622, E11.628, E11.63, E11.630, E11.638, E11.64, E11.641, E11.649, E11.65, E11.69, E11.8, E11.9, E13
Chronic Liver Disease/Cirrhosis	K70, K70.0, K70.1, K70.10, K70.11, K70.2, K70.3, K70.30, K70.31, K70.4, K70.40, K70.41, K70.9, K72.1, K72.10, K72.11, K72.9, K72.90, K72.91, K73, K73.0, K73.1, K73.2, K73.8, K73.9, K74, K74.0, K74.1, K74.2, K74.3, K74.4, K74.5, K74.6, K74.60, K74.69, K74.5, K75.9, K76.1, K76.9
Dementia	F01, F01.5, F01.50, F02, F02.8, F02.80, F02.81, F03, F03.9, F03.90, F03.91, F04
Frailty	R54, R53.0, R53.1, R53.2, R53.8, R53.81, R53.82, R41.81, R53.83
Inability to Walk	R26, R26.0, R26.1, R26.2, R26.8, R26.9, R27, R27.0, R27.8, R27.9, R29.3, R29.4, R29.6
Depression	F32, F32.0, F32.1, F32.2, F32.3, F32.4, F32.5, F32.9, F33, F33.0, F33.1, F33.2, F33.3, F33.4, F33.40, F33.41, F33.42, F33.8, F33.9
Malnutrition	E40, E41, E42, E43, E44, E44.0, E44.1, E45, E46
Obesity	E66, E66.0, E66.01, E66.09, E66.1, E66.2, E66.3, E66.8, E66.9
